# Supplementary figures and images for: Tissue resident cells differentiate S. aureus from S. epidermidis via IL-1β following barrier disruption in healthy human skin
Source: PLoS Pathog. 2024 Aug 29;20(8):e1012056. doi: 10.1371/journal.ppat.1012056 (PMC11389914; doi:10.1371/journal.ppat.1012056)

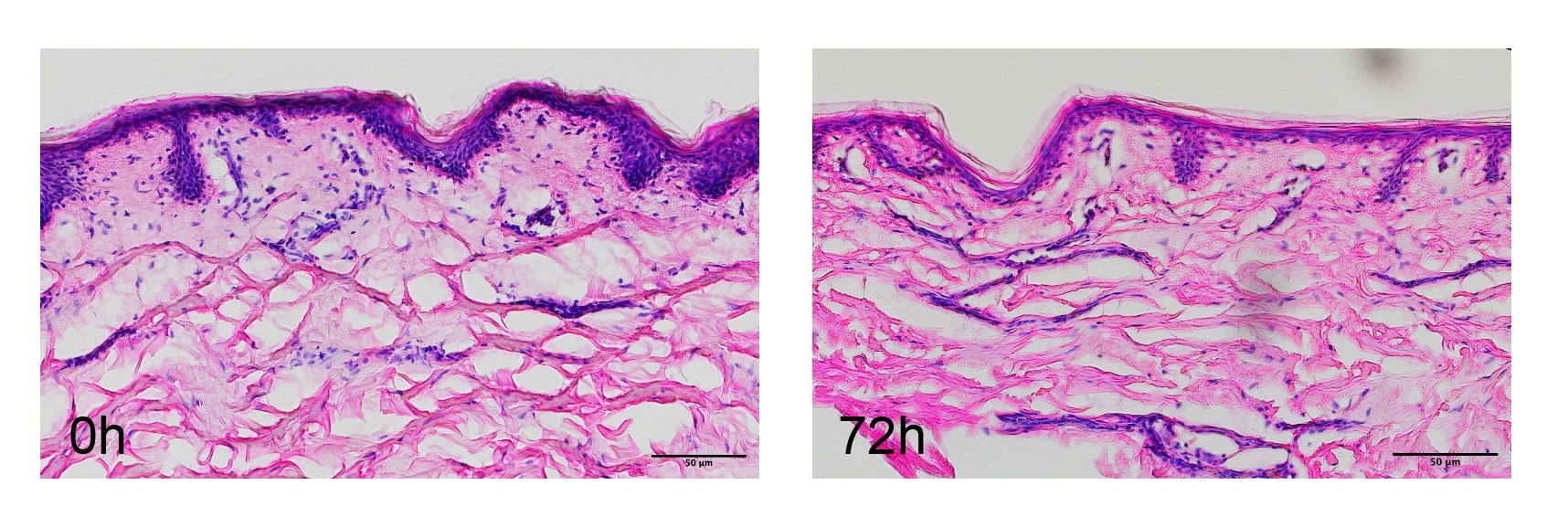

Supplement: S1 Fig — H&E staining of human skin cross sections 0 h and 72 h after ex vivo culture Scale bar = 50μm. (TIF) [file ppat.1012056.s001.tif]

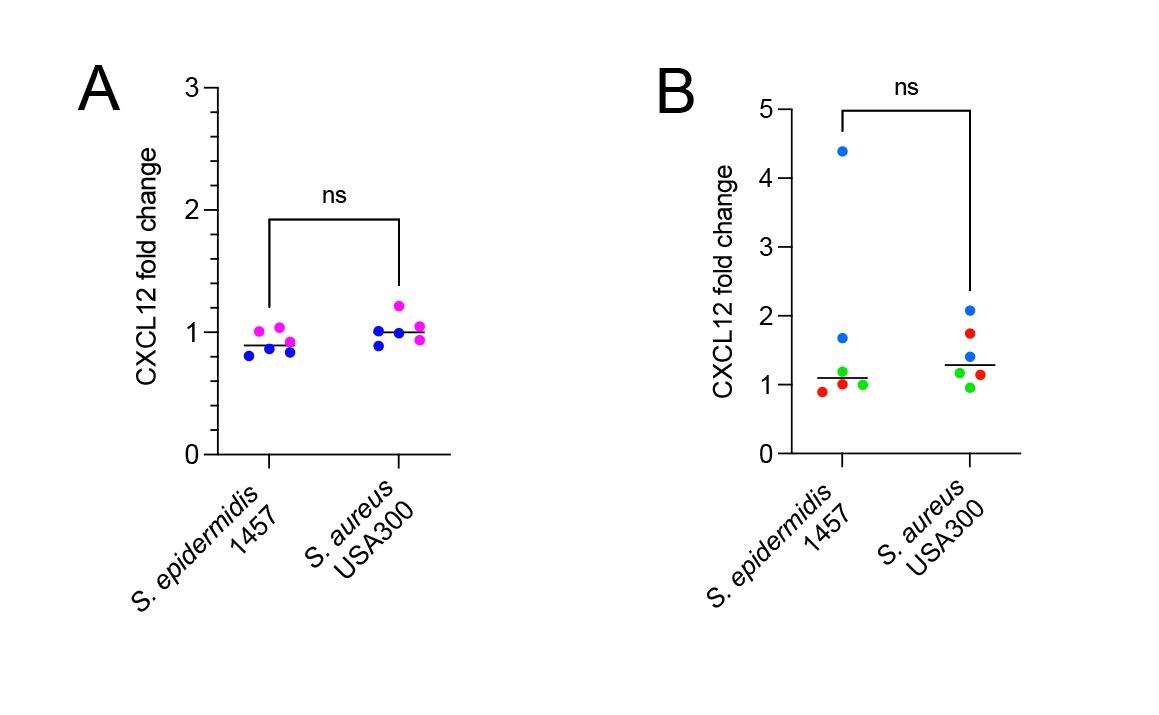

Supplement: S2 Fig — ELISA CXCL12 of intact (A) and disrupted (B) human skin homogenates 48h after colonization with S. epidermidis 1457 and S. aureus USA300, displayed as x-fold change over uninfected controls. Dots represent individual data points, colours differentiate skin donors (n = 2–3). Significance determined by unpaired t-test with Welch’s correction. (TIF) [file ppat.1012056.s002.tif]

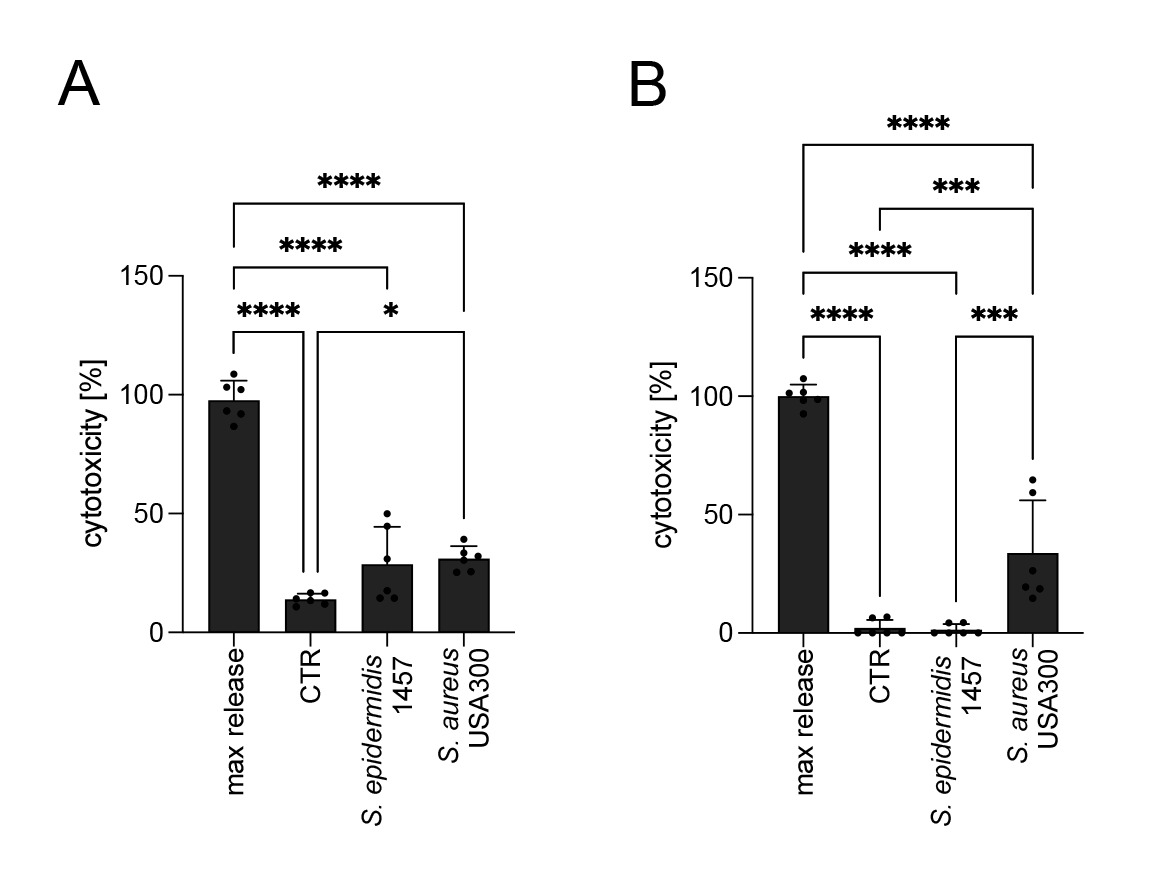

Supplement: S3 Fig — Cytotoxicity of keratinocytes (A) and fibroblasts (B) infected for 3 h with S. epidermidis 1457 or S. aureus USA300, measured after 24 h by LDH assay. Bars = means ± SD; dots represent individual data points (n = 3, with 2 technical replicates). Significance determined by ordinary one-way ANOVA with Tukey’s correction for multiple comparisons. Significance is denoted by * p ≤ 0.05, *** p ≤ 0.001 or **** p ≤ 0.0001. (TIF) [file ppat.1012056.s003.tif]

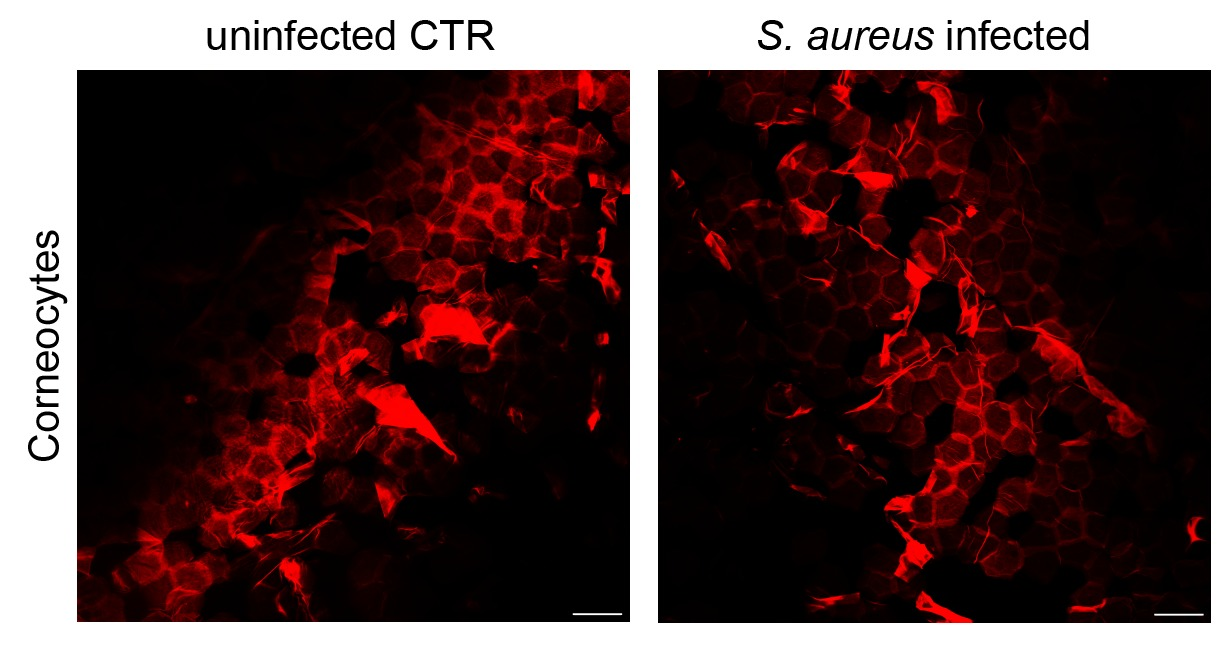

Supplement: S4 Fig — Microscopic detection of intracellular S. aureus GFP in cell monolayers of tape-stripped human corneocytes; uninfected CTR vs 3h infection. Autofluorescence staining of corneocytes (red). Scale bars = 50μm. (TIF) [file ppat.1012056.s004.tif]

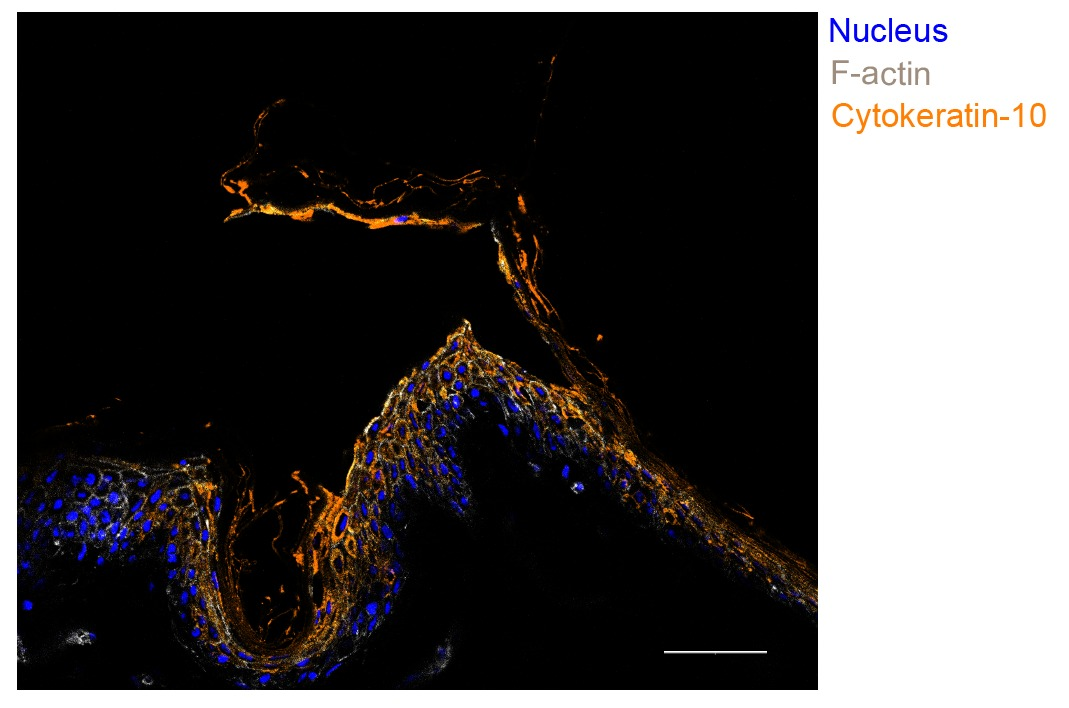

Supplement: S5 Fig — Representative microscopy image of disrupted stratum corneum by scratching the surface of human skin biopsies. Immunofluorescent staining of keratinocytes in stratum spinosum and granulosum expressing cytokeratin-10 (orange), f-actin (white), nuclei (blue). Scale bar = 50μm. (TIF) [file ppat.1012056.s005.tif]

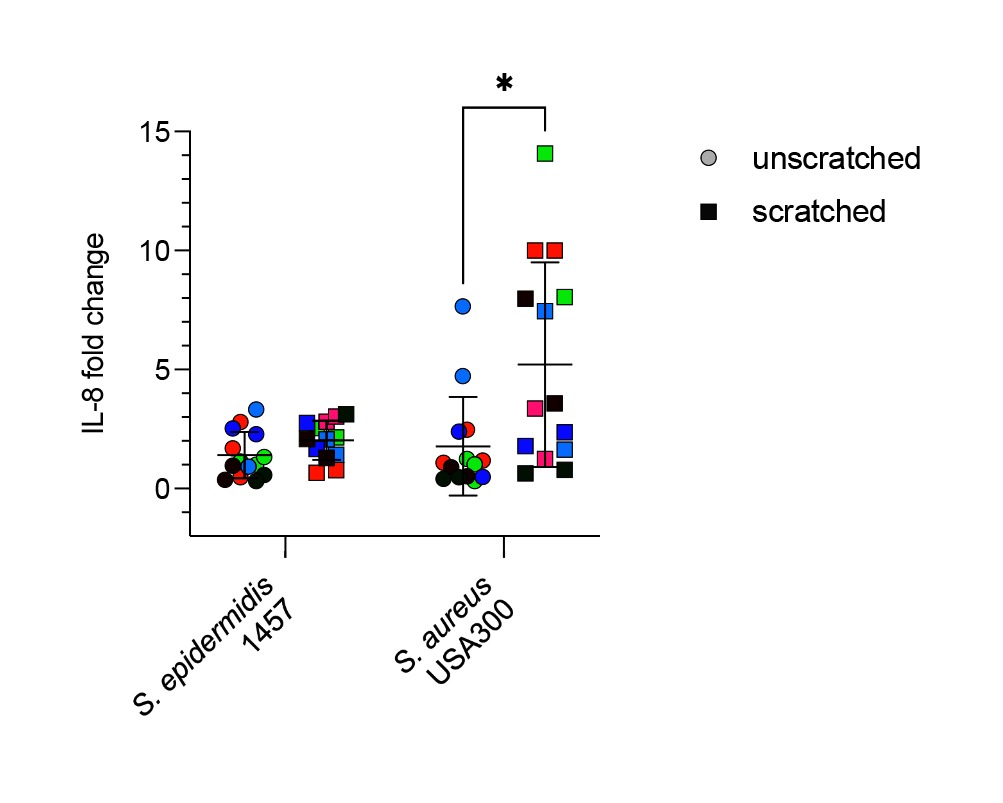

Supplement: S6 Fig — Comparison of data shown individually in Figs 1 and 3. ELISA IL-8 of human skin homogenates 48 h after colonization with S. epidermidis 1457 and S. aureus USA300, displayed as x-fold change over uninfected controls. Dots represent individual data points, colours differentiate skin donors (n = 6–7). Significance determined by unpaired t-test with Welch’s correction and Holm-Šídák’s multiple comparison test. Significance is denoted by * p ≤ 0.05. (TIF) [file ppat.1012056.s006.tif]

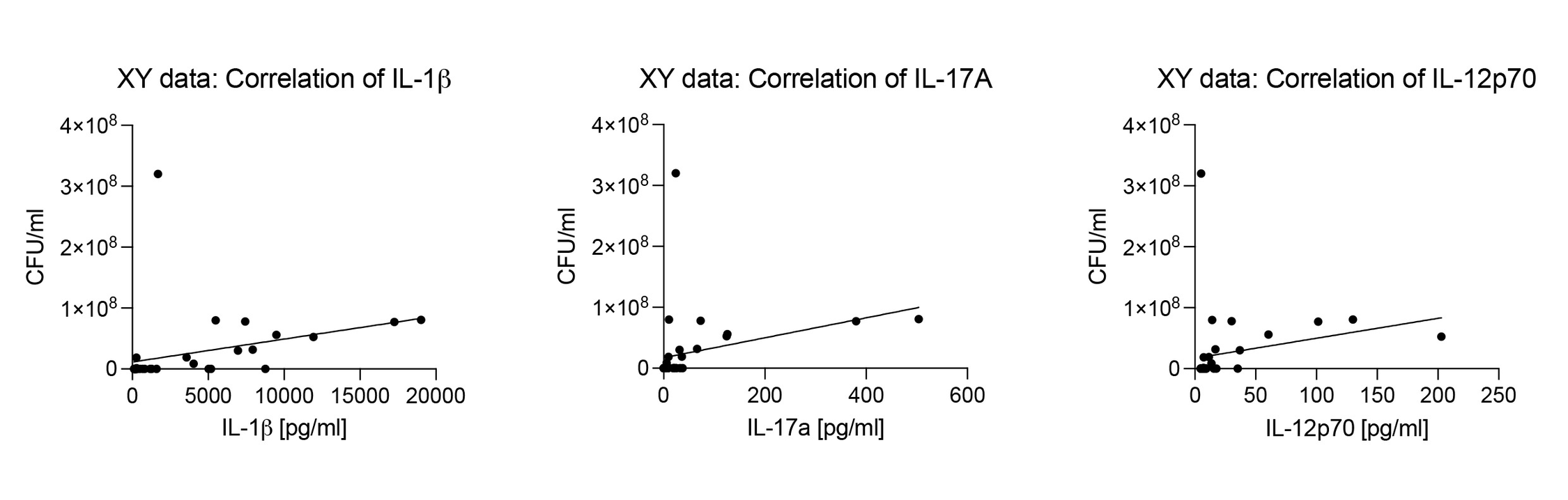

Supplement: S7 Fig — Correlation between CFU/ml and concentration of IL-1β, IL-17A and IL-12p70 in human skin biopsies 48h p.i. A two tailed Spearman’s rank-order correlation shows a positive relationship between CFUs and IL-1β (r = 0.7112, p<0.0001), IL-17A (r = 0.5390, p = 0.0010) and IL-12p70 (r = 0.4958, p = 0.0029). (TIF) [file ppat.1012056.s007.tif]

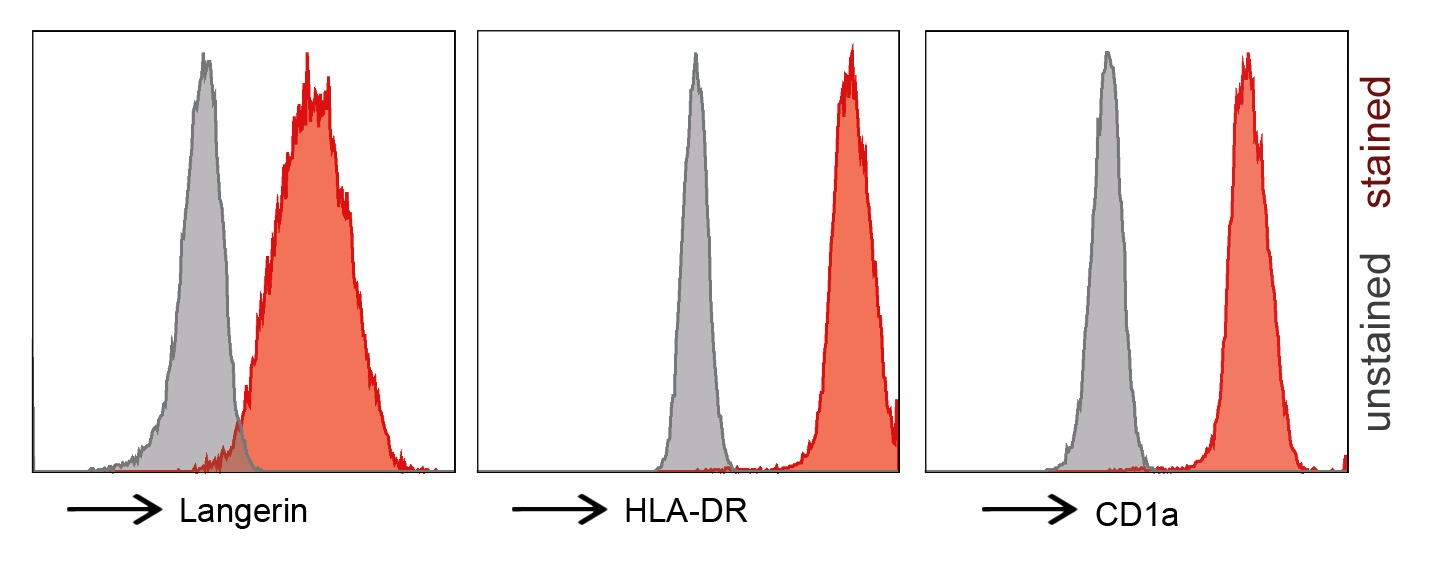

Supplement: S8 Fig — Histograms of stained (red) MUTZ-LCs expressing langerin (CD207), CD1a and HLA-DR vs unstained MUTZ-LCs (grey) analyzed by FACS. (TIF) [file ppat.1012056.s008.tif]

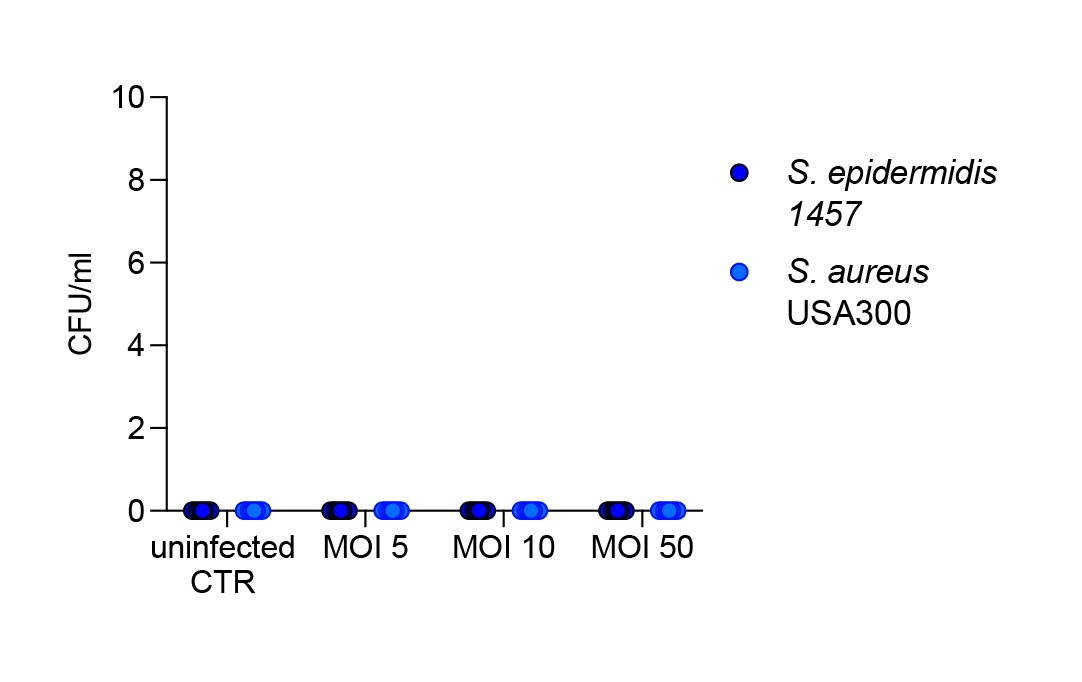

Supplement: S9 Fig — Intracellular CFU counts of S. epidermidis 1457 and S. aureus USA300 3 h p.i. of MUTZ-LC. Dots show individual data points (n = 3; 2 technical replicates). (TIF) [file ppat.1012056.s009.tif]

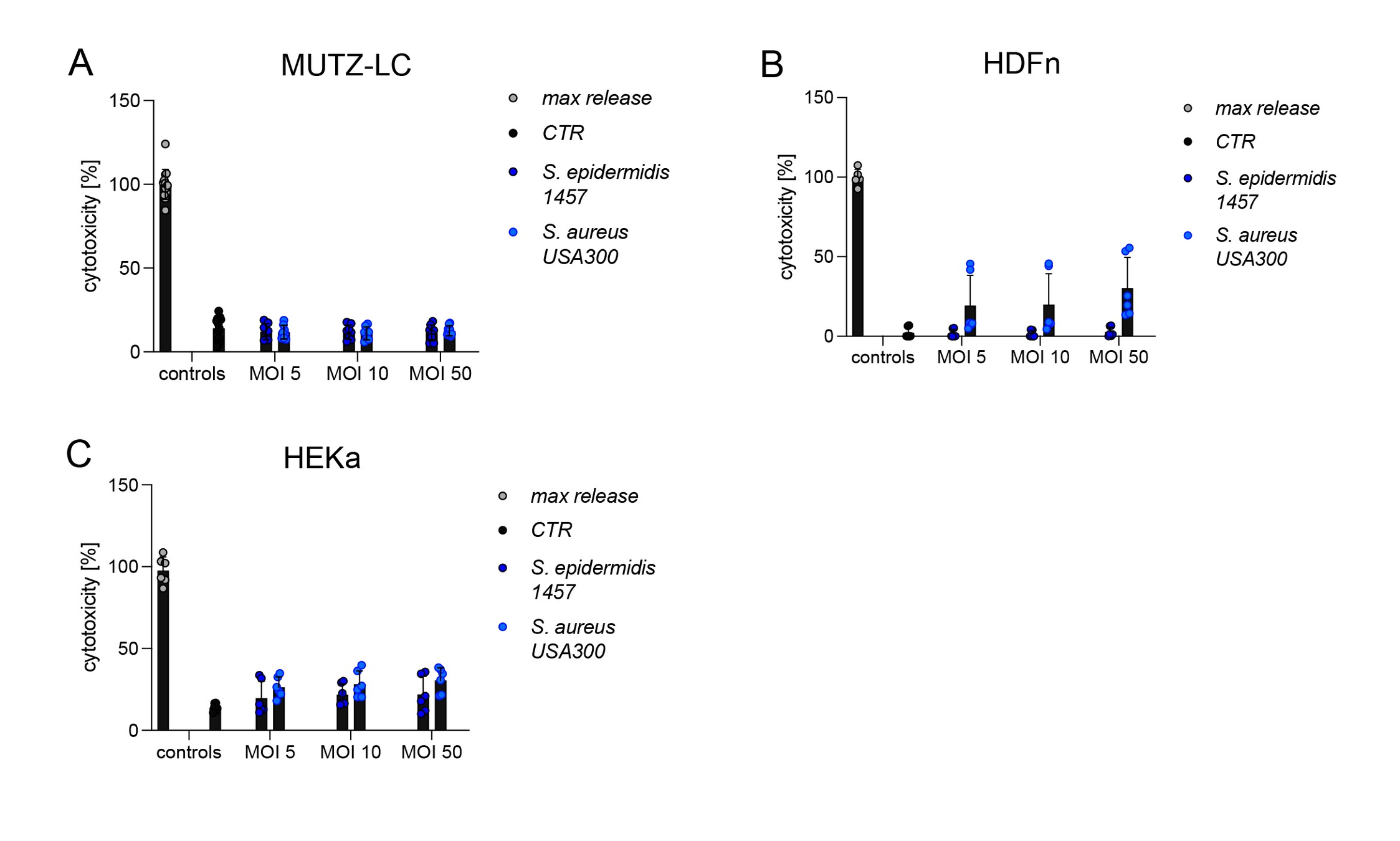

Supplement: S10 Fig — Cytotoxicity of MUTZ-LC (A), fibroblasts (B) and keratinocytes (C) infected for 3h with S. epidermidis 1457 or S. aureus USA300 measured after 24 h by LDH assay. Bars = means ± SD; dots represent individual data points (n = 3, with 2 technical replicates). Significance determined by Ordinary one-way ANOVA with Tukey’s correction for multiple comparisons. Significance is denoted by * p ≤ 0.05. (TIF) [file ppat.1012056.s010.tif]

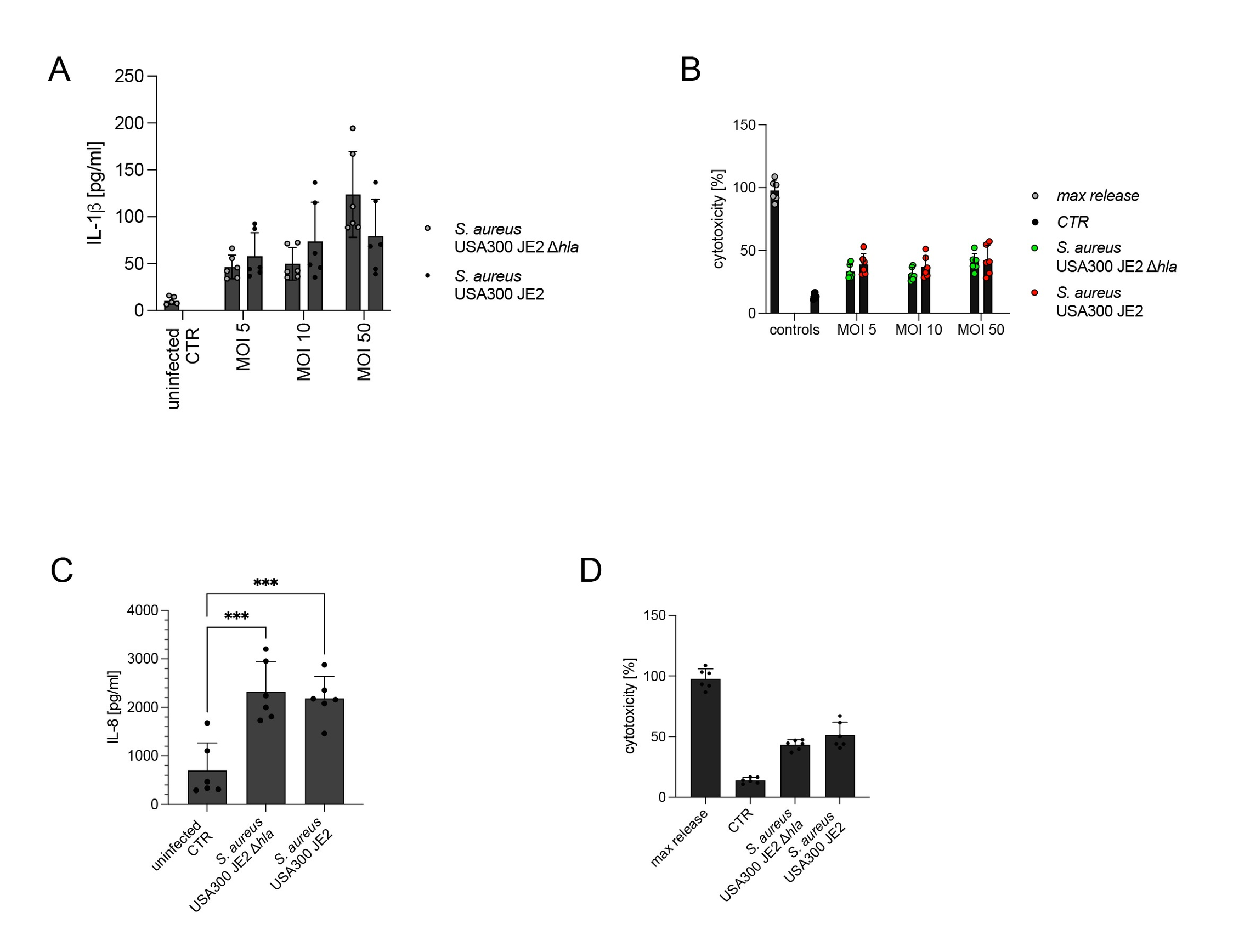

Supplement: S11 Fig — (A) IL-1β measured by ELISA in HEKa supernatants at 24 h p.i. after 3 h infection with S. aureus USA300 JE2 Δhla or S. aureus USA300 JE2. Bars represent means ± SD, dots show individual data points (n = 3, with 2 technical replicates). (B) Cytotoxicity of keratinocytes infected for 3h with S. aureus USA300 JE2 Δhla or S. aureus USA300 JE2, measured after 24 h by LDH assay. Bars = means ± SD; dots represent individual data points (n = 3, with 2 technical replicates). (C) IL-8 measured by ELISA in HEKa supernatants at 24 h p.i. after 3 h infection with MOI100 S. aureus USA300 JE2 Δhla or S. aureus USA300 JE2. Bars represent means ± SD, dots show individual data points (n = 3, with 2 technical replicates). (D) Cytotoxicity of keratinocytes infected for 3 h with MOI100 S. aureus USA300 JE2 Δhla or S. aureus USA300 JE2, measured after 24 h by LDH assay. Bars = means ± SD; dots represent individual data points (n = 3, with 2 technical replicates). (TIF) [file ppat.1012056.s011.tif]
